# Regulation of *Hoxb4 *induction after neurulation by somite signal and neural competence

**DOI:** 10.1186/1471-213X-9-17

**Published:** 2009-02-25

**Authors:** Gayana S Amirthalingam, Sara Howard, Susana Alvarez, Angel R de Lera, Nobue Itasaki

**Affiliations:** 1 Division of Developmental Neurobiology, MRC National Institute for Medical Research, The Ridgeway, London, NW7 1AA, UK; 2 Department of Organic Chemistry, Faculty of Chemistry, Universidad de Vigo, 36310 Vigo, Spain

## Abstract

**Background:**

While the body axis is largely patterned along the anterior-posterior (A-P) axis during gastrulation, the central nervous system (CNS) shows dynamic changes in the expression pattern of *Hox *genes during neurulation, suggesting that the CNS refines the A-P pattern continuously after neural tube formation. This study aims at clarifying the role of somites in up-regulating *Hoxb4 *expression to eventually establish its final pattern and how the neural tube develops a competence to respond to extrinsic signals.

**Results:**

We show that somites are required for the up-regulation of *Hoxb4 *in the neural tube at the level of somites 1 to 5, the anterior-most domain of expression. However, each somite immediately adjacent to the neural tube is not sufficient at each level; planar signaling is additionally required particularly at the anterior-most segments of the expression domain. We also show that the dorsal side of the neural tube has a greater susceptibility to expressing *Hoxb4 *than the ventral region, a feature associated with dorsalization of the neural tube by BMP signals. BMP4 is additionally able to up-regulate *Hoxb4 *ventrally, but the effect is restricted to the axial levels at which *Hoxb4 *is normally expressed, and only in the presence of retinoic acid (RA) or somites, suggesting a role for BMP in rendering the neural tube competent to express *Hoxb4 *in response to RA or somite signals.

**Conclusion:**

In identifying the collaboration between somites and neural tube competence in the induction of *Hoxb4*, this study demonstrates interplay between A-P and dorsal-ventral (D-V) patterning systems, whereby a specific feature of D-V polarity may be a prerequisite for proper A-P patterning by *Hox *genes.

## Background

The anterior-posterior (A-P) identity of the body axis at the level of the hindbrain and the spinal cord is largely dependent upon the regulated expression of *Hox *gene clusters [1,2]. At early embryogenesis, *Hox *genes are up-regulated sequentially in the epiblast and establish their ordered expression patterns along the A-P axis [3,4]. They also play an instructive role in distributing cells in an ordered manner along the A-P axis during ingression of epiblast cells [5]. As a consequence, *Hox *gene expression exhibits nested patterns in the paraxial mesoderm as well as in the neuroepithelium. One unique feature of conferring A-P identity by *Hox *genes is that these nested expression patterns display sharp anterior boundaries, creating codes of expression along the A-P axis [6,7]. For example, expression of paralogue 4 *Hox *genes, such as *Hoxb4*, have an anterior-most limit at the rhombomere 6/7 boundary, while the anterior most limit of paralogue 5 genes lies at the rhombomere 7/8 boundary. Thus rhombomere 7 is defined as a *Hox *paralogue 4 positive and *Hox *paralogue *5 *negative segment. As evidence of this code-dependent positional identity, null mutant mice of *Hox *genes exhibit the loss of a segmental identity only of the anterior-most domain of the gene expression [8-11]. Hence, regulation of *Hox *expression at the anterior-most domain is the most crucial step in the process of conferring A-P identity.

**Figure 1:**
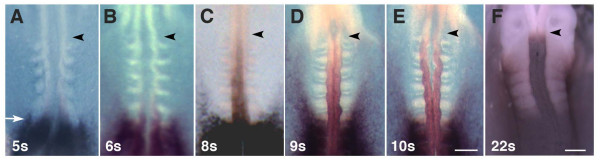
**Up-regulation of *Hoxb4 *expression in the neural tube at 5 to 22 somite stages**. (A-F) Whole-mount embryos stained for *Hoxb4 *at 5 to 22 somites stages (5S to 22S) as indicated. Up to the 5 somite stage, the anterior boundary of expression is located at the 6^th ^somite level both in the neural tube and paraxial mesoderm (A, arrow). At 6–10 somite stages, expression in the neural tube exclusively extends anteriorly while mesodermal expression remains at the same level (B-E). At the 22 somite stage, the expression shows its anterior most boundary at the rhombomere 6/7 level (F). Arrowheads indicate the prospective rhombomere 6/7 boundary. Scale bars; 200 μm.

While the expression of *Hox *genes begins at the primitive streak stage, cells are not committed to express specific *Hox *genes and the pattern does not strictly follow the cell lineage. Instead, the expression patterns of many *Hox *genes display dynamic changes during neurulation. In addition to *Hoxb4*, as described below in detail (Fig. 1), *Hoxb1*, *b3 *[3] and *b9 *[12] in chick and *Hoxb5, b6 and b8 *in mouse [13,14] have been shown to exhibit dynamic alterations in their expression patterns during axis elongation before the final pattern is established.

What is the possible factor responsible for the dynamic change in *Hox *gene expression in the neural tube? One strong candidate is the influence from flanking somites. It has been shown in chick embryos that transposition of regions of the neural tube along the A-P axis results in the reprogramming of *Hox *codes [15,16]. Furthermore, somites have been shown to be able to up-regulate *Hoxb4 *when grafted ectopically in regions that do not normally express *Hoxb4 *[16,17]. Similar results were obtained in zebrafish embryos, where grafting of non-axial mesoderm causes transformation of forebrain to a hindbrain character [18]. These observations led to the idea that the neural tube undergoes continual assessment of its environmental signals in order to establish the correct pattern of *Hox *gene expression in the CNS after neural tube formation [16].

Retinoic acid (RA) is the most likely molecule responsible for the up-regulation of *Hox *genes by the somites. An enzyme retinaldehyde dehydrogenase-2 (RALDH2), which converts the inactive form retinaldehyde to RA, is expressed in the somites from early stages of development [19-21]. RA appears to be abundant in the neural tube as well as in the somites [22]. Cyp1B1, another RA synthesizing enzyme, is also expressed in somites during early embryogenesis [23]. In mice, at least, RALDH2 seems to be the main RA synthesizing enzyme in early embryogenesis at 7.5 dpc and 8.5 dpc, since the RA-responsive transgene RARE-hsp-LacZ [24] does not show expression in *RALDH2*-/- embryos at these stages except in the eye [25]. RA deficiency caused either by genetic deletion of *RALDH2 *in mice [25,26] or by placing quail hens on a RA-deficient diet [27] results in defects in axial development and patterning. The defects do not span the whole *Hox*-territory; rather, the defect is restricted to the posterior hindbrain (rhombomeres 4–8 including the level of somites 1–5), demonstrating that this region requires correct RA levels [28,29].

To account for the complex organization of *Hox *genes by signaling mechanisms, a number of models have been proposed. For example, individual *Hox *genes have specific retinoic acid response elements (RAREs) with different sensitivities to RA, thereby allowing each *Hox *gene to be controlled differently depending on the concentration of RA [30]. Another example of differential regulation of *Hox *genes is by FGF signaling, where some *Hox *genes have the binding sequence for a downstream transcription factor, Cdx, in their enhancers [31-33]. In addition to the above, it is likely that there are more mechanisms that are responsible for establishing the correct A-P pattern, such as the duration of exposure to signals, the degree of dependence on signals, the involvement of planar signaling, and the competence of the neural tube to respond to signals. This study aims at clarifying two issues. First, to what extent does endogenous *Hox *expression depend upon the somite signal? Second, what determines the competence of the neural tube to respond to the somite signal? In order to address these questions, *Hoxb4 *has been chosen as a model because the dynamic changes in its expression pattern occur at the stages when the tissues involved are accessible for refined dissections. The chick explant culture system was employed to identify tissue interactions that are responsible for the up-regulation of *Hoxb4 *expression in the neural tube.

## Results

### The dynamics of *Hoxb4 *expression in the developing chick neural tube

We first investigated in detail the changes in the *Hoxb4 *expression pattern that occur after neurulation. *Hoxb4 *is first detectable at the full streak stage (stage 4 of Hamburger and Hamilton, HH 4) [34] and the anterior-most boundary resides at the level of the future 6^th ^somite both in the mesoderm and the neural tube until the 5 somite stage (HH 8^+^) [3] (Fig. 1A). This level is about 5 somite segments more posterior than the final anterior-most boundary of neural tube expression, the rhombomere 6/7 boundary, which corresponds to the anterior edge of the 1^st ^somite. During somite stages 6 to 10 (HH 9^- ^to 10), which is approximately a difference of 6 hours, the expression pattern rapidly changes exclusively in the neural tube; the domain here extends anteriorly while mesodermal expression remains at the same level (Fig. 1B–E). Expression extends until it finally establishes its anterior most limit at the future rhombomere 6/7 boundary. This does not involve cell movement as cells in the neural tube maintain their relative positions to the flanking somite at these stages [35]. During somite stages 10–20 (HH 10–13), the expression becomes stronger while the domain remains unchanged (data not shown). By the 22 somite stage (HH 14), the rhombomere 6/7 boundary is formed and the *Hoxb4 *domain is clearly defined (Fig. 1F).

### Signals from the mesoderm are necessary for the initial up-regulation of *Hoxb4*

**Figure 2:**
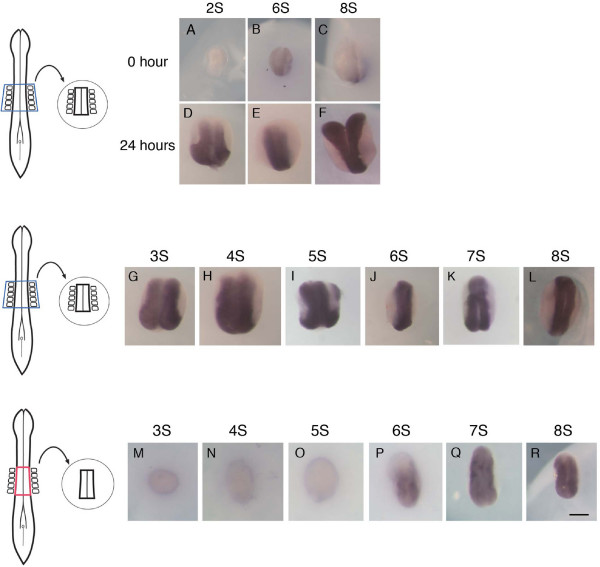
**The explant culture system recapitulates the initiation of *Hoxb4 *expression in the neural tube and reveals a stage-dependent requirement of somites**. (A-F) As schematized in the left of the panel, explants of neural tube were taken with flanking somites at the level of somites 1 to 5, from embryos at 2 (A, D), 6 (B, E) or 8 (C, F) somite stages, and either fixed immediately (A-C) or cultured for 24 hours (D-F). Immediately fixed explants show no or faint expression of *Hoxb4 *depending on the stage of explanting, reflecting the normal expression (A-C, n = 3/3 at each stage). After 24 hours of incubation, the explants show strong expression of *Hoxb4 *throughout the length of the neural tube (D-F, n = 6/6 at each stage). Indicated above each panel are the somite stages (S) at which explants were dissected. (G-R) Neural tube explants were taken either with (G-L) or without (M-R) flanking somites, at stages between 3 and 8 somites (3S to 8S) and cultured for 24 hours. All explants that included somites strongly express *Hoxb4 *homogenously in the neural tube (G-L, n = 6/6 at each stage). When somites are removed, *Hoxb4 *expression is absent in explants from 3 to 5 somite stages (M-O, n = 6/6 at each stage). In explants taken at the 6 somite stage, weak expression is observed (P, n = 8/8). It is at the 7 and 8 somite stages that the explants express *Hoxb4 *strongly (Q, R, n = 6/6 at each stage). Scale bar; 200 μm.

Although it has been previously shown that somites are capable of inducing *Hoxb4 *in ectopic locations [16], it is not known whether somites are required for the endogenous up-regulation of *Hoxb4*. If so, at which stage are they required and are they sufficient for the correct patterning of the neural tube in normal development? In order to address these questions, the somite level 1–5 region was analyzed using the explant culture system. First, in order to test whether the initiation of *Hoxb4 *expression in the neural tube is recapitulated in the explant culture system, the neural tube at somite level 1–5 including flanking somites, as well as surface ectoderm, notochord and endoderm, were dissected from embryos between 2 and 8 somite stages. It was found that, in all explants dissected at the above stages, *Hoxb4 *expression was up-regulated in the neural tube along the axial length after 24 hours of culture (Fig. 2A–F). Presence or absence of notochord did not have an effect on the result (data not shown). Next, in order to investigate the requirement of somites, the neural tube was dissected without somites (Fig. 2M–R) and compared to a neural tube that included flanking somites (Fig. 2G–L). The results show that neural explants taken between somite stages 3–5 did not show any up-regulation of *Hoxb4 *after 24 hours (Fig. 2M–O). Some weak expression was observed in the posterior half when the explant was taken at the 6 somite stage (Fig. 2P). Conversely, from the 7 somite stage onwards, the removal of somites did not affect the level of expression (Fig. 2Q, R), indicating that somites are not required at these stages.

### At a given A-P level, signals from the somites are not sufficient to up-regulate *Hoxb4 *expression

**Figure 3:**
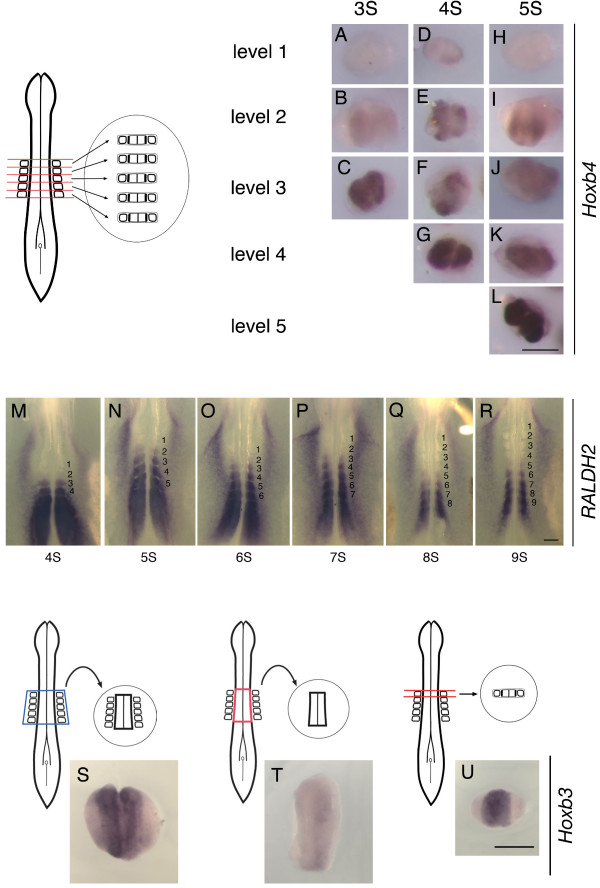
**Signaling of each somite to the adjacent neural tube**. (A-L) Neural tube and somites were dissected at each somite level as shown in the scheme and cultured for 24 hours. Embryos used as donors were at the 3 (A-C), 4 (D-G) or 5 (H-L) somite stage (3S, 4S, 5S) as indicated above the column. Level 1–5 indicates the origin of explants at each somite level. The expression of *Hoxb4 *is graded with posterior regions displaying the strongest expression. Note that somite level 1 neural tube does not exhibit any *Hoxb4 *expression (A, D, H). The number of cases is shown in Additional file 3. (M-R) Expression pattern of *RALDH2 *in chick embryos at the 4 to 9 somite stages (4S to 9S). Somite numbers are indicated in each figure to identify the level. (S, T) Neural tube explants either with (S) or without (T) flanking somites at the level of 1 to 5 somites, taken at the 5 somite stage, cultured for 24 hours and assayed for *Hoxb3 *expression. Up-regulation of *Hoxb3 *requires the presence of flanking somites, in the same manner as that of *Hoxb4 *(S, n = 5/5; T, n = 6/6; compare with Fig. 2I, O). (U) Neural tube and somites were dissected at the level of somite 1 at the 5 somite stage and cultured for 24 hours to assay for *Hoxb3 *expression. Up-regulation of *Hoxb3 *is seen (n = 6/6, compare with Fig. 3H where *Hoxb4 *is negative). Scale bars; 200 μm.

The requirement of somites in the above experiment led us to consider whether each somite is solely responsible for inducing *Hoxb4 *at the same level of neural tube. In other words, at a particular level of the neural tube, are the adjacent somites sufficient to pattern this level or not? In order to test this, the neural tubes of 3 to 5 somite stage embryos were dissected at each somite level including somites (Fig. 3A–L and Additional File 3). After 24 hours, it was found that posterior levels (somite levels 4 and 5) strongly expressed *Hoxb4 *in the neural tube (Fig. 3G, K, L), whereas neural tube at somite levels 2 and 3 exhibited weaker *Hoxb4 *expression (Fig. 3B, C, E, F, I, J). The neural tube at somite level 1 displayed no expression (Fig. 3A, D, H). These results suggest the following: (1) Vertical signals (from somite to the adjacent neural tube at the same axial level) alone are not sufficient, and hence planar communication and signaling in the neural tube along the A-P axis are to be considered. (2) Different axial levels exhibit different degrees of vertical signaling, either by posterior somites producing a greater signal than anterior ones or by different axial levels of the neural tube responding differently to the somite signal, or both. There are two pieces of evidence that support the idea that somites send different degrees of signal strength to the neural tube. Firstly, ectopic somite grafting showed that the more posterior somites have a stronger inducing capability in the adjacent neural tube [16]. Second, *RALDH2 *is expressed strongly in posterior somites whereas there is very little or no expression in somite 1 [20] (Fig. 3M–R). In fact, the ability of each somite to induce *Hoxb4 *expression in the adjacent neural tube, both ectopically [16] and at endogenous locations (Fig. 3A–L), coincides spatially and temporally with the graded expression of *RALDH2 *in somites (Fig. 3M–R). However, there is some discrepancy in considering *RALDH2 *as the sole factor responsible for up-regulating *Hoxb4*: At the 8 somite stage when *Hoxb4 *is being up-regulated but is not yet being expressed in anterior regions (Fig. 1C), *RALDH2 *has already been down-regulated at most of the somite 1–5 levels (Fig. 3Q). In addition, while the *Hoxb4 *expression domain extends anteriorly during the course of development, *RALDH2 *expression is down-regulated in an anterior to posterior direction (Fig. 3M–R). Another RA synthesizing enzyme, Cyp1B1, is expressed in somites at young stages without any significant down-regulation [23]. However, despite the expression of Cyp1B1 in somites, anterior somites such as 1 and 2 failed to fully up-regulate *Hoxb4 *in the adjacent neural tube in the above experiment (Fig. 3A, B, D, E, H, I). These results led us to further analyze the role of somites in up-regulating *Hoxb4 *in the neural tube.

The above experiments suggest that the ability of somites to induce *Hox *genes in the adjacent neural tube is not simply determined by the production of RA. Other factors to be considered include the identity of the *Hox *gene, the relative timing of up-regulation of *Hox *genes and RA production, and the A-P position within the *Hox *gene expression domain. In order to address these, we used *Hoxb3 *as another *Hox *marker. *Hoxb3 *has an anterior-most expression domain at the rhombomere 5/6 boundary and establishes its final expression pattern at the 9 somite stage, slightly earlier than *Hoxb4 *[3]. We first confirmed using the explant culture method that up-regulation of *Hoxb3 *requires flanking somites at the 5 somite stage, in a similar manner to *Hoxb4 *(Fig. 3S, T). By dissecting somite level 1 neural tube along with flanking somites, it was observed that somite 1 is sufficient to up-regulate *Hoxb3 *in the adjacent neural tube (Fig. 3U). Hence, the 1^st ^somite is not incapable of inducing *Hox *genes; the failure to up-regulate *Hoxb4 *in somite level 1 neural tube is not due to the specific feature of the 1^st ^somite; rather, different signal strengths are required for the up-regulation of different *Hox *genes. Nevertheless, the result of dissecting neural tube and flanking somites at each of the somite levels 1 to 5 (Fig. 3A–L) is in clear contrast to the result of explanting the neural tube and somites as a whole (Fig. 2G–I), where the neural tube displays homogenous expression along its axial length. These data suggest that vertical signals alone are not sufficient and planar communication or signaling is required.

### Involvement of planar signaling

**Figure 4:**
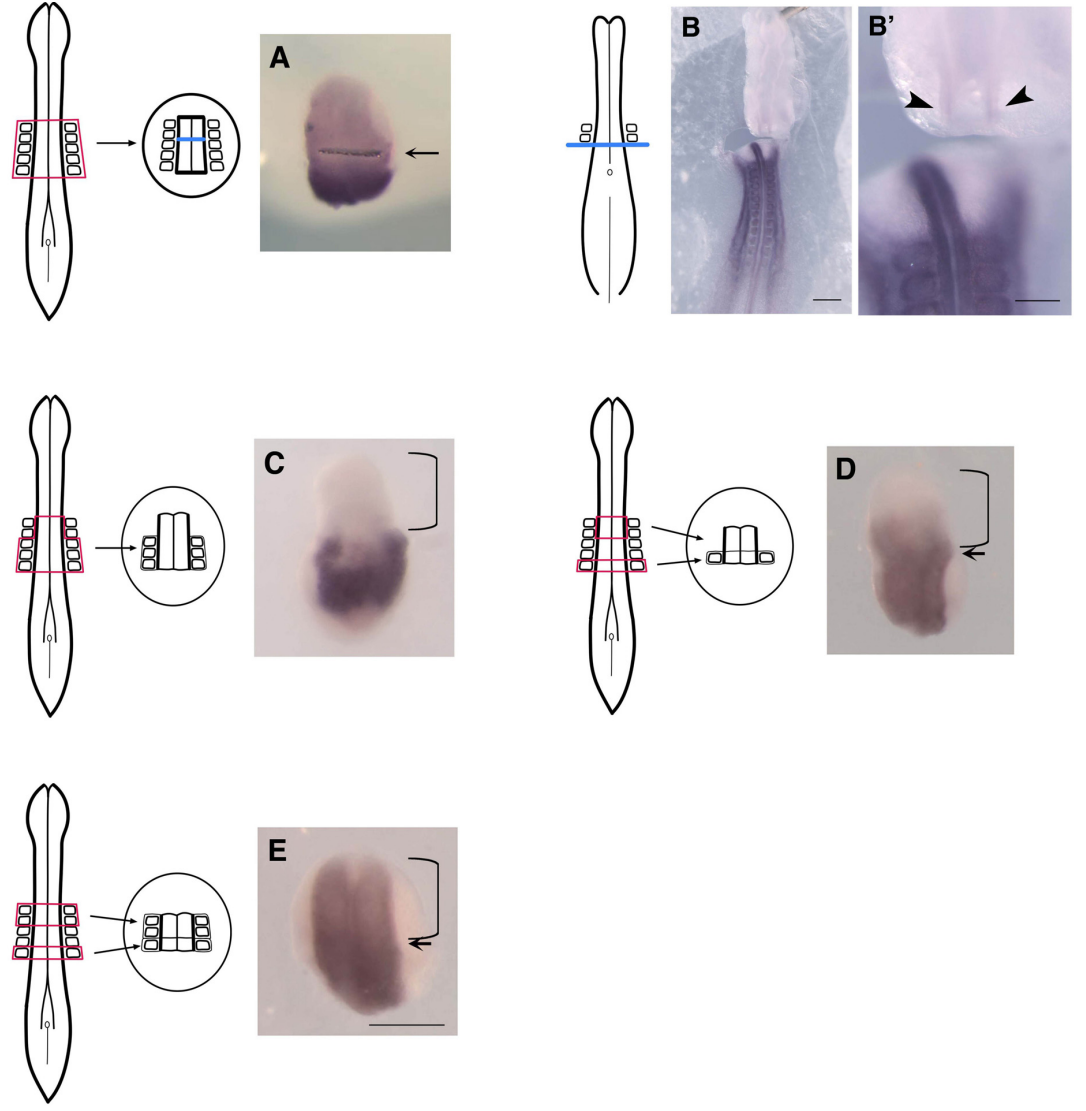
**Requirement for planar signaling in the up-regulation of *Hoxb4***. (A) Neural tube from somite level 1–5 was dissected from 5 somite stage embryos with adjacent somites and a foil barrier was placed in the neural tube at the level between somites 2 and 3 (arrow). Following a 24-hour culture, no up-regulation of *Hoxb4 *was seen anterior to the barrier (n = 7/10. 3/10 showed weak expression anterior to the barrier). (B) *Ex ovo *cultured chick embryo inserted with a foil barrier placed across the whole tissues at the level posterior to the 2^nd ^somite, at the 2 somite stage. Following a 24-hour culture, *Hoxb4 *expression in the anterior side of the barrier is inhibited (arrowheads in B'). Other cases of this experiment together with controls are shown in Additional File 1. (C) Neural tube from somite level 1–5 was dissected from 5 somite stage embryos. Somites 1 and 2 were removed while somites 3–5 were kept. *Hoxb4 *expression is absent in the anterior neural tube (bracket) (n = 5/6. 1/6 showed weak expression in the neural tube level 1 and 2). (D, E) Neural tube at somite level 1 and 2 was dissected either without (D) or with (E) somites and fused with a somite level 5 neural tube that included flanking somites. Bracket marks the somite level 1–2 region, whereas arrow marks the somite level 2/5 border. Following a 24-hour culture, (D) shows weak expression in the somite level 2 region but not in the somite level 1 region (n = 8/10. 2/10 showed no expression). (E) shows expression in both the level 1 and 2 regions (n = 5/5). Scale bars; 500 μm (B), 200 μm (B', E).

To directly test the possible involvement of planar signaling, attempts were made to block planar signaling in the neural tube explant. Neural tube from a 5 somite stage embryo was dissected from somite level 1–5 including adjacent somites. A foil barrier was placed in the neural tube at the level of the somite 2/3 boundary (but not in between the somites) (Fig. 4A). Positioning the barrier in such a manner would block the possible occurrence of planar signals, yet still allow the transmission of vertical signals at each level. Following a 24-hour culture, *Hoxb4 *failed to be up-regulated significantly in the neural tube at somite level 1 and 2 (i.e. the region anterior to the barrier). This implies that the placement of a barrier did block transmission of signals travelling anteriorly through the plane of the neural tube. Weak expression present in the region anterior to the barrier might be attributed to vertical signals coming from somites immediately adjacent to the neural tube.

Barrier-placing experiments were further conducted using whole embryos at the 2 somite stage, by placing a barrier posterior to the 2^nd ^somite (future somite 2/3 boundary). The barrier was placed across the neural tube as well as all other tissues including somites. Embryos were incubated *ex ovo *on albumen-agar plates [36], which helped stable positioning of the barrier. After 24–26 hours of incubation, *Hoxb4 *induction on the anterior side of the barrier was found to be clearly blocked, either completely (n = 4/12) or to significantly low levels compared to the posterior side of the barrier (n = 7/12) (Fig. 4B, Additional file 1). This result is consistent with the above experiment of explant culture suggesting planar signaling; however, it is not in agreement with the study by Gaunt and Strachan (1994) where the *Hoxd4 *expression domain was shown to 'spread forward' regardless of physical barrier after 24 hours of culture. The reason for this discrepancy is not clear. One possible explanation is that *ex ovo *culture may cause delays in chick development and *Hoxd4/b4 *might be up-regulated after a longer incubation, although embryos incubated *ex ovo *in the same condition without barriers showed a clear up-regulation of *Hoxb4 *as normal (Additional file 1). However, there was one case showing strong *Hoxb4 *induction anterior to the barrier (n = 1/12, Additional file 1), suggesting that the results may vary among the cases. Nonetheless, both our explant and *in vivo *studies suggest the requirement of tissue continuity in up-regulating *Hoxb4 *in the neural tube, suggesting involvement of planar signaling. The planar signaling may be required for the initial extension in expression, and/or the maintenance of the expression initiated by signals from flanking somites.

Given the importance of planar signaling in the neural tube, it was next asked whether anterior somites are required for the planar signaling or not. Somites 1 and 2, although unable to up-regulate *Hoxb4 *in the adjacent neural tube in a vertical manner (Fig. 3), are still required for *Hoxb4 *expression, since removal of somites 1 and 2 from the explant of somite level 1–5 abolished up-regulation of *Hoxb4 *in the anterior neural tube (Fig. 4C, compare with Fig. 2I). This could be because somites are either required for instructive vertical signals or for sending permissive cues that allow the anterior neural tube to respond to planar signals. We have noticed that the neural tube and somites from level 4 or 5 cause a stronger induction in the level 1 neural tube compared to those from more anterior levels such as levels 1–3 (Additional file 2). We therefore designed experiments where a neural explant dissected at somite levels 1–2 (without flanking somites) was combined with a level 5 neural tube with flanking somites (Fig. 4D). Following a 24 hour culture, significant up-regulation of *Hoxb4 *was seen in the anterior neural tube in an area approximately one somite diameter in length (Fig. 4D). Hence, the neural tube is able to up-regulate *Hoxb4 *in the absence of flanking somites when combined with a more posterior neural tube with somites. In a similar experiment where the neural explant of somite level 1–2 was combined with a level 5 neural tube, where all levels included somites, it was noted that up-regulation of *Hoxb4 *was fully extended to the anterior-most end of the explant (Fig. 4E), despite that these anterior somites are not capable of sufficiently up-regulating *Hoxb4 *in the adjacent neural tube (Fig. 3H, I). These results demonstrate that somites 1 and 2 are required for up-regulation of *Hoxb4 *in the neural tube, at least in part to assist in the response of the neural tube to planar signaling, suggesting a synergistic effect between planar and vertical signals.

### Dorso-ventral difference of *Hox *gene expression

**Figure 5:**
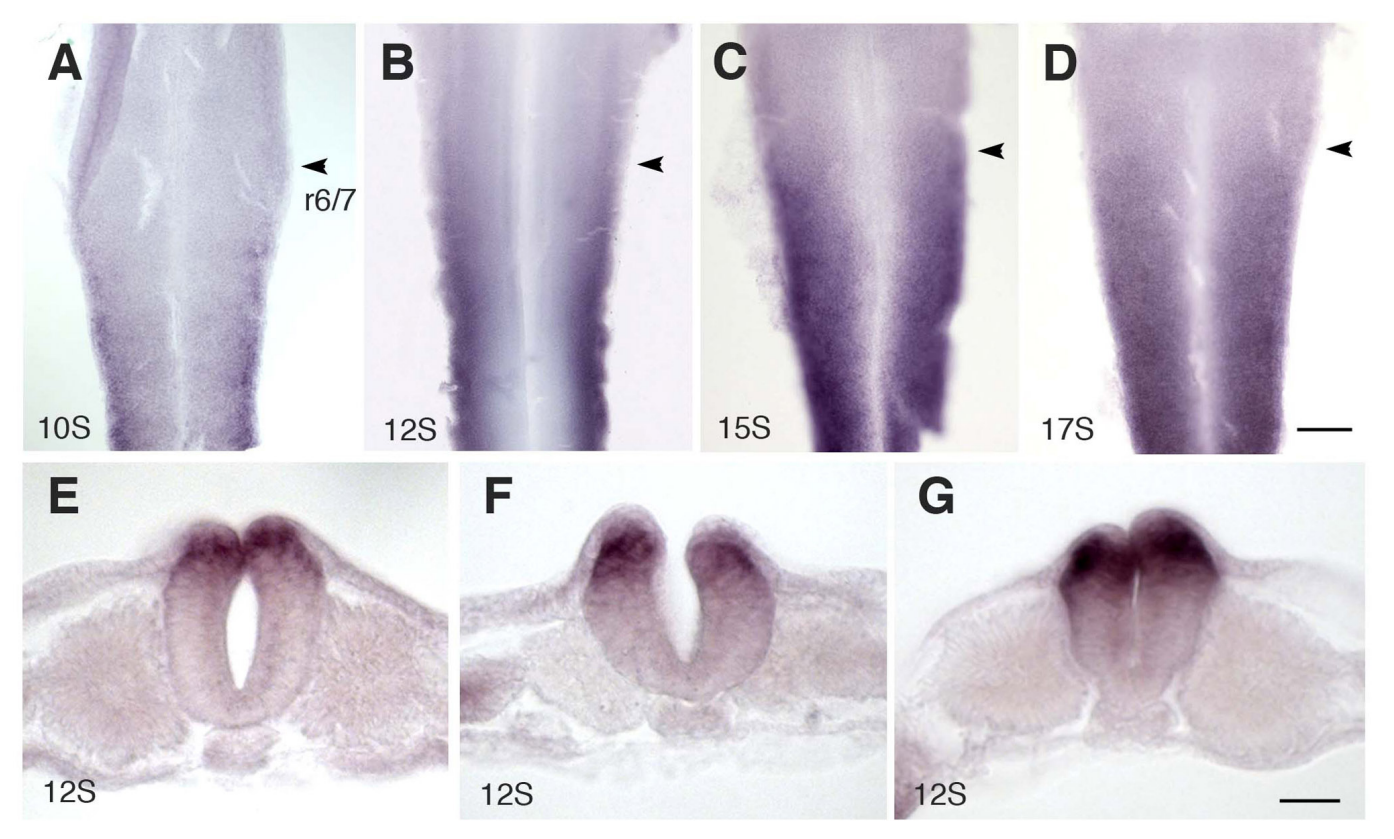
**The dorsal side of the neural tube precedes the ventral side in up-regulating *Hoxb4 *expression**. (A-D) A series of chick neural tube from 10 (A), 12 (B), 15 (C) and 17 (D) somite stage embryos, stained for *Hoxb4*. The neural tube is flat-mounted by opening it from the dorsal side which is now located laterally. Arrowheads indicate the rhombomere 6/7 boundary (r6/7). At the 10 somite stage, when the anterior-most expression has not reached r6/7 (see also Fig. 1E), the up-regulation is restricted to the dorsal-most edge of the neural tube (A). At the 12 somite stage, a distinct D-V difference is seen (B). By the 15 somite stage, this D-V difference becomes less apparent in accordance with the expression becoming stronger, although the most-ventral region of the neural tube does not express *Hoxb4 *(C). (D) is at the 17 somite stage when the expression is mostly homogeneous except in the floor plate region. Scale bar; 100 μm. (E-G) Transverse sections of a 12 somite chick embryo stained for *Hoxb4*, at the level of the 1^st ^somite (E), 5^th ^somite (F) and 10^th ^somite (G). Dorsally localized expression is seen in the neural tube at all axial levels. Scale bar; 50 μm.

The above results collectively demonstrate that vertical signals do not sufficiently establish the correct expression pattern of *Hoxb4*. This led us to consider the possibility of a factor located within the neural tube that may be able to affect or modulate the response of the neural tube to the somite signal. In line with investigating factors that facilitate the neural tube to express *Hoxb4*, we have noticed that the neural tube shows a considerable difference in *Hox *gene expression along the dorso-ventral (D-V) axis, where the dorsal side expresses more strongly than the ventral side during the course of up-regulation at 10–12 somite stages (Fig. 5A, B). Transverse sections clearly show that up-regulation of *Hoxb4 *begins at the dorsal-most side of the neural tube, with this D-V difference being seen at all axial levels at these stages (Fig. 5E–G and data not shown). It is noticeable that the dorsal side of the neural tube, which is where *Hoxb4 *is strongly expressed initially, does not necessarily have close contact with the flanking somites (Fig. 5E–G), suggesting that up-regulation in the dorsal side cannot be explained solely by somitic signals. At the 15 somite stage, the D-V difference is less evident, except that the floor plate does not express *Hoxb4 *(Fig. 5C). At the 17 somite stage, the neural tube shows homogeneous expression of *Hoxb4 *along the D-V axis except in the floor plate (Fig. 5D).

There is additional evidence indicating that the dorsal side of the neural tube precedes the ventral side in *Hoxb4 *expression: When the neural tube is translocated posteriorly, the graft up-regulates *Hoxb4 *in response to the new posterior environment. In this situation, the dorsal side of the graft expresses *Hoxb4 *earlier than the ventral side [16]. In another situation, where somites are grafted anteriorly into the pre-otic region causing ectopic induction of *Hox *genes, *Hoxb4 *up-regulation can be seen predominantly in the dorsal edge of the neural tube, which is particularly the case when anterior somites with weak inductive abilities are used [16]. Furthermore, during the normal course of development (HH 18–20), there is a transient up-regulation of *Hoxb4 *in the dorsal rim of rhombomere 6 while the anterior-most limit of the main expression domain is at the rhombomere 6/7 boundary [16]. These observations collectively imply that the dorsal side of the neural tube might have a greater susceptibility to expressing *Hox *genes than the ventral region.

### BMP signals are involved in up-regulating *Hoxb4 *in the neural tube

**Figure 6:**
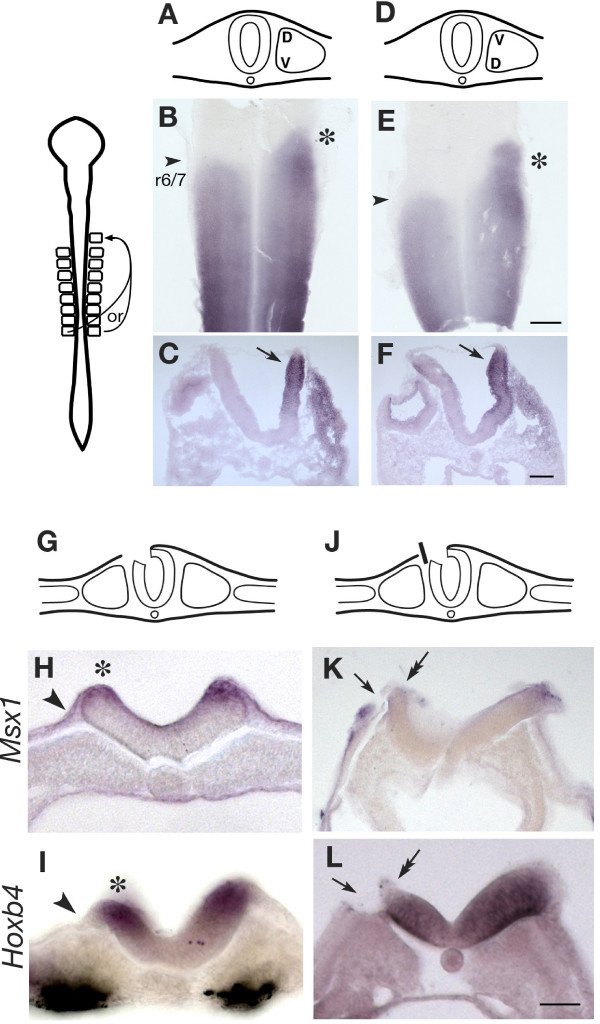
**Dorsal precedence of *Hoxb4 *expression is associated with the dorsalized feature of the neural tube**. (A-F) Grafting of the 8^th ^somite anteriorly to the pre-otic region induces ectopic up-regulation of *Hoxb4*. In (A-C), the D-V orientation of the somite was kept as normal. In (D-F), the somite graft was taken from the contralateral side and rotated 180°, which reversed the D-V orientation of the graft but kept the medio-lateral orientation the same. (B) and (E) are flat-mounts of neural tube at HH 16, showing the dorsal neural tube at lateral sides. Asterisks show ectopic induction of *Hoxb4 *by grafted somites. Arrowheads show rhombomeres 6/7 boundary (r6/7). Scale bar; 200 μm. (C) and (F) are transverse sections of similar embryos at HH 15, at the level of rhombomere 4–6. In both cases, strongest induction is observed at the dorsal neural tube (arrows) and graded ventrally, regardless of the orientation of grafted somites (n = 5/5 for each orientation). Scale bar; 100 μm. (G-L) Ablation of the surface ectoderm and dorsal neural tube. In (G-I), the roof plate of the left side of the neural tube was ablated at the 3–4 somite stage from somite level 1–5, which was accompanied by removal of surface ectoderm just covering the neural tube as shown in the scheme (G). Six hours later, the surface ectoderm had regenerated (arrowheads in H, I), and both *Msx1 *(H, n = 8/8) and *Hoxb4 *(I, 8/10) expression had been restored (asterisks in H, I). In (J-L), the roof plate and surface ectoderm were similarly ablated but a foil barrier was placed to prevent the ectoderm from regenerating, as illustrated in (J). In these embryos, the surface ectoderm maintained discontinuity with the dorsal neural tube throughout the following incubation (arrows in K, L). These embryos failed to up-regulate *Msx1 *(K, n = 4/5) and *Hoxb4 *(L, n = 5/7) at the dorsal neural tube (double arrows in K, L). Scale bar; 100 μm.

The above observations prompted us to investigate the cause of the dorsal precedence, since this may provide us with a key to understanding the susceptibility of the neural tube to expressing *Hoxb4*. There are two possibilities that may account for this D-V difference in *Hox *expression: (1) The dorsal region of the somite is sending a stronger signal to the neural tube than the ventral region. (2) The somite is sending a uniform strength of signal to the neural tube along its D-V axis, but the dorsal neural tube is more responsive to this signal than the ventral neural tube. To investigate whether the dorsal region of the somite is transmitting a stronger signal to the neural tube than the ventral region, ectopic induction of *Hoxb4 *was reassessed using dorso-ventrally rotated somites. A single somite was taken from a posterior level of a 8–9 somite stage embryo (i.e. a somite with a *Hoxb4 *inducing ability in the pre-otic region) [16] and ectopically grafted, while either preserving or reversing the D-V orientation, into the region adjacent to prospective rhombomere 5 of a stage-matching host embryo (where *Hoxb4 *is not normally expressed but is competent to respond to somite signals resulting in induction of *Hoxb4*). Following a 24–33 hour culture, when the ectopic induction was clearly observed, expression of *Hoxb4 *in the rhombomere 5 region was not significantly different between the cases of rotated and non-rotated somites (Fig. 6A–F). Therefore, it is unlikely that the dorsal region of the somite is sending a stronger signal than the ventral region; rather the somite conveys a uniform strength of signal along its D-V axis. This raises the possibility that the neural tube itself is responding differently along its D-V axis to the somite signal, where the dorsal neural tube is more responsive to the somite signal than ventral regions.

An obvious feature of the dorsal neural tube is its contact with the surface ectoderm and subsequent expression of BMP ligands and other dorsal neural tube markers such as Msx1/2 and Pax7 [37]. It has been shown that a neural explant induces dorsal markers Msx1/2 in response to contact with the surface ectoderm [38]. In fact, transverse sections of an embryo at the 12 somite stage revealed that up-regulation of *Hoxb4 *appears to occur most strongly in the region where the neural tube is in contact with the surface ectoderm (Fig. 5E–G). Hence, an experiment was conducted to determine whether *Hoxb4 *expression is up-regulated in response to the dorsalizing signal from the ectoderm *in vivo*. The dorsal neural tube (approximately 10–15% of the neural tube) was ablated at the level of prospective somites 1–5 on one side together with the surface ectoderm immediately overlying the neural tube (Fig. 6G). In some cases, a foil barrier was placed at the edge of the surface ectoderm to prevent it from regenerating and fusing to the dorsal neural tube (Fig. 6J). The ablation was conducted on 3 to 4 somite stage embryos, after which they were incubated until the 10–12 somite stage. Without a foil barrier, the surface ectoderm rapidly regenerated within 6 hours following ablation and formed a continuous epithelial layer with the ablated end of the neural tube. In these cases, up-regulation of *Msx1 *was seen at the dorsal tip of the ablated side of the neural tube (Fig. 6H), as was *Hoxb4 *(Fig. 6I). In contrast, in cases where regeneration of the surface ectoderm was inhibited by the barrier, neither *Msx1 *(Fig. 6K) nor *Hoxb4 *(Fig. 6L) were up-regulated. This suggests that up-regulation of *Hoxb4 *in the dorsal neural tube is associated with contact with the surface ectoderm and subsequent dorsalization of the neural tube.

### BMP4 requires RA signalling to up-regulate *Hoxb4*

**Figure 7:**
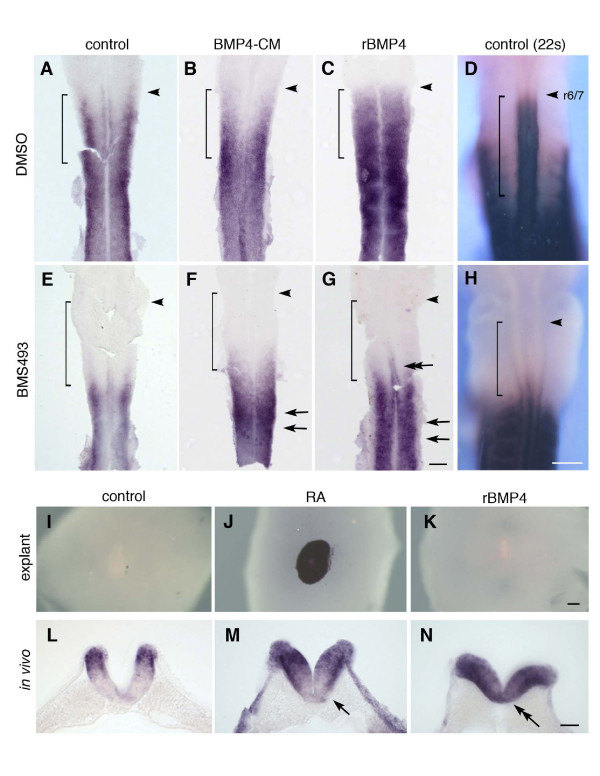
**BMP4-dependent *Hoxb4 *up-regulation requires RA at the level of somites 1–5**. (A-C, E-G) Flat-mounted neural tube from embryos treated with DMSO (A-C) or BMS493 (E-G) from 4 to 5 somite stages. Embryos were cultured either in the control medium (A, E), in the conditioned medium of *BMP4*-transfected cells (BMP4-CM; B, F) or in the presence of recombinant BMP4 (rBMP4; C, G) until they reached the 10 to 12 somite stages. In the absence of BMS493 (A-C), additional BMP4 causes ventral expansion of *Hoxb4 *expression (B, C, n = 12 for each) compared to control (A). In the presence of BMS493 (E-G), *Hoxb4 *is not up-regulated at the level of somites 1 to 6 regardless of additional BMP4 (F, G, n = 16 for each), except that a slight up-regulation is observed at the ventral neural tube by rBMP4 (G, double arrow). At more posterior levels, ventral expansion is induced by additional BMP4 (arrows in F, G). Arrowheads indicate rhombomere 6/7 boundary (r6/7). Brackets indicate the level of somites 1 to 6. Scale bar; 100 μm. (D, H) Whole mount chick embryos cultured *ex ovo *with DMSO (D) or BMS493 (H) from the 2 to 4 somite stages and incubated until the 22 somite stage. *Hoxb4 *expression is specifically blocked in the neural tube at the level of somites 1 to 6 (bracket) (n = 4). Scale bar; 200 μm. (I-K) Neural tube explants at the level of somites 1 to 5, without flanking somites, cultured in the presence of RA or recombinant BMP4 (rBMP4) for 24 hours and analyzed for *Hoxb4 *expression. While RA sufficiently up-regulates *Hoxb4 *(J), rBMP4 does not do so (K) (n = 3 for each). Scale bar; 200 μm. (L-N) Transverse sections of embryos cultured *ex ovo *in the control medium (L), with RA (M) or with recombinant BMP4 (N). Sections are at the level of somite 3 to 4. Control embryos show *Hoxb4 *expression localized to the dorsal side (n = 5/5). Exogenous RA causes a stronger and broader expression in the dorsal half of the neural tube, but the expression domain does not expand to ventral regions (M, arrow) (n = 6/7. 1/7 showed a expansion close to the floor plate). Recombinant BMP4 causes an expansion of *Hoxb4 *expression to the ventral side of the neural tube (double arrow) (n = 4/6. 2/6 showed only a weak expansion to the floor plate). Scale bar; 100 μm.

Since the dorsalization of the neural tube by the surface ectoderm is known to be mediated by BMP signaling [38], we next directly tested whether BMP signaling up-regulates *Hoxb4*. Embryos at 4–5 somite stages were cultured *ex ovo *[39] using either conditioned medium of HEK293 cells transfected with *BMP4 *or recombinant BMP4 (see Materials and Methods) and analyzed at 10 to 12 somite stages when the D-V difference of *Hoxb4 *expression was prominent in a control condition. Embryos treated with BMP4 displayed a significant ventral expansion of *Hoxb4 *expression in the neural tube, while maintaining the anterior-most limit at the rhombomere 6/7 boundary (Fig. 7A–C). This difference was not prominent at later stages when control embryos also express *Hoxb4 *ventrally (data not shown). Hence the result suggests that BMP4 may facilitate the neural tube to up-regulate *Hoxb4 *ventrally.

Since RA is able to up-regulate the expression of *Hoxb4 *and is known as the likely candidate for the somite-derived signals, the possible mechanism for up-regulating *Hoxb4 *expression by BMP4 was further examined to determine if BMP4 is capable of up-regulating *Hoxb4 *independently of RA or whether BMP4 requires RA in order to up-regulate *Hoxb4*. With the aim of blocking RA signaling, embryos at 4–5 somite stages were cultured *ex ovo *in the presence of a RA receptor antagonist, BMS493 [40]. BMS493-treated embryos failed to up-regulate *Hoxb4 *in the neural tube at the level of somites 1–6, when observed at both the 10–12 and 22 somite stages (Fig. 7A, E and 7D, H, respectively). This was consistent with the result seen in vitamin A deficient quail and *RALDH2*-/- mouse embryos [25-28,41,42]. In the presence of BMS493, additional BMP4 protein did not up-regulate *Hoxb4 *expression at the level of somites 1 to 6 (Fig. 7F, G). Therefore it is suggested that, during the course of *Hoxb4 *up-regulation at the level of somites 1 to 6, RA is absolutely required and BMP4 cannot compensate for its absence. This was supported by explant culture experiments, where the neural tube at the level of somites 1 to 5 was dissected without flanking somites and cultured in the presence of RA or BMP4 (Fig. 7I–K). While exogenous RA sufficiently up-regulated *Hoxb4 *expression in the neural tube explant (Fig. 7J), BMP4 was not able to do so (Fig. 7K), suggesting that BMP4 cannot exert its function to up-regulate *Hoxb4 *in the absence of somites. Hence, while RA plays an instructive role in the up-regulation of *Hoxb4*, BMP4's role is likely to be permissive rather than instructive.

We further investigated whether RA is able to promote ventral expansion of *Hoxb4 *expression in a similar manner to BMP4 *in vivo*. While BMP4 showed clear up-regulation in the ventral side of the neural tube (Fig. 7L, N), exogenous RA only enhanced the dorsally dominant *Hoxb4 *expression and did not show up-regulation in the ventral neural tube as significantly as BMP4 did (Fig. 7M). The result that exogenous RA cannot up-regulate *Hoxb4 *in the ventral neural tube while BMP4 can, underscores a distinct role of BMP signaling in the *in vivo *context. Collectively, these results suggest a two-phase model in establishing *Hoxb4 *expression in the axial level of somite 1–6. First *Hoxb4 *is up-regulated at the dorsal neural tube by signals from the surface ectoderm, likely mediated by BMP or TGFβ signaling (Fig. 6G–L). RA is required for the dorsal patterning process (Wilson et al., 2004), and hence this initial phase likely employs both signals. Second, *Hoxb4 *expression spreads more ventrally, which can be promoted by exogenous BMP4 but not by RA. However, this process does not occur in the absence of RA, at least at the somite level 1 to 6. It is not clear in the experiment of Figure 7 using BMS493 *in vivo*, whether the requirement of RA is only in the initial step at the dorsal side, or also in the up-regulation at the ventral side. However, the result that after removal of dorsal neural tube, the remaining ventral neural tube shows *Hoxb4 *expression in a comparable manner to the control side (Fig. 6L) suggests that RA/somite up-regulates *Hoxb4 *at the ventral side independently of the preceding dorsal expression. Given the direct role of RA on the *Hoxb4 *enhancer [30], the data suggest distinct functions of RA and BMP signals for up-regulating *Hoxb4 *in the ventral neural tube, where RA provided by somites functions as an essential signal, while BMP4 functions as a factor facilitating the neural tube to respond to the RA/somite signal.

It was noted that, in the neural tube at the level posterior to the 7^th ^somite, BMP4 is able to up-regulate *Hoxb4 *even in the presence of BMS493 (Fig. 7E–G, arrows). This axial level does not require RA signaling for *Hoxb4 *expression [25-28,41,42]. Hence there remains a possibility that BMP signals may be able to up-regulate *Hoxb4 *independent of RA.

### Dorsalization of the neural tube precedes up-regulation of *Hoxb4*

**Figure 8:**
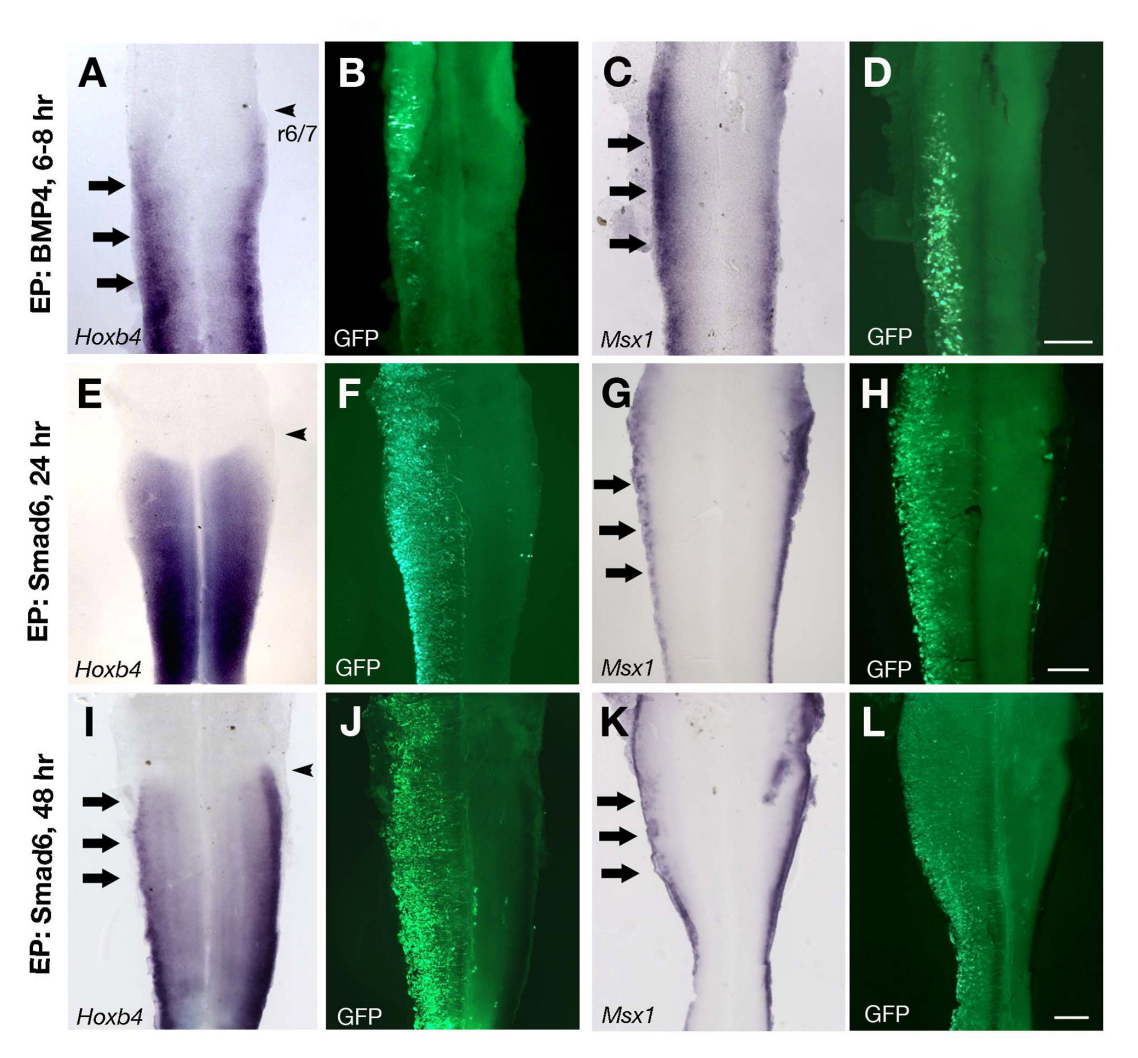
**Dorsal expression of *Hoxb4 *is preceded by dorsalization of the neural tube by BMP signals**. (A-D) Flat-mounted neural tubes from embryos electroporated with *BMP4*, incubated for 6–8 hours and assayed for *Hoxb4 *(A) and *Msx1 *(C). Electroporation of *BMP4 *results in a ventral expansion of *Hoxb4 *(A, n = 25/30) and *Msx1 *(C, n = 14/15) within 6–8 hours. Scale bar; 100 μm. (E-H) Electroporation of *Smad6 *followed by 24 hours of incubation does not cause any obvious changes in *Hoxb4 *expression pattern (E, n = 23/25. 2/25 showed down-regulation). *Msx1 *expression is down-regulated at the electroporated side (G, n = 19/20). Scale bar; 200 μm. (I-L) Electroporation of *Smad6 *followed by 48 hours of incubation causes down-regulation of both *Hoxb4 *(I, n = 19/21) and *Msx1 *(K, n = 18/20). Arrows indicate changes in the expression compared to the contralateral side. (B, D, F, H, J, L) shows electroporated, GFP-positive cells in (A, C, E, G, I, K), respectively. Arrowheads in (A, E, I) show the rhombomere 6/7 boundary (r6/7). Scale bar; 200 μm.

We further examined whether the up-regulation of *Hoxb4 *by BMP4 is a direct effect of activation of BMP pathway, or as a consequence of the dorsalized feature of the neural tube. Consistent with the result of embryo cultures with exogenous BMP4, electroporation of *BMP4 *in the neural tube at the 5 somite stage followed by 6–8 hours of incubation caused a noticeable up-regulation of *Hoxb4 *expanded toward the ventral side (Fig. 8A, B). This was accompanied by up-regulation of other dorsal neural markers such as *Msx1 *(Fig. 8C, D) and *Pax7 *(data not shown). Electroporation of a GFP construct did not show any changes (data not shown). Hence, the D-V pattern of the neural tube has already been altered by the time we observe the changes in the *Hoxb4 *expression. Next, the same stage of neural tube was electroporated with *Smad6 *to test the requirement of BMP signals for *Hoxb4 *expression. Smad6 blocks transduction of BMP and TGFβ signals at the intracellular level [43]. Smad6 successfully inhibited *Hoxb4 *expression at the dorsal side of the neural tube (Fig. 8I, J). However, the down-regulation was not seen at 6 (data not shown) or 24 hours (Fig. 8E, F) but at 48 hours of incubation (Fig. 8I, J), which was presumably due to the late onset of exogenous Smad6 expression, which might not be prompt enough to override the endogenous programme. In fact, Smad6 electroporation caused down-regulation of *Msx1/2 *not at 6 hours, but after 24 (Fig. 8G, H and data not shown) and 48 hours (Fig. 8K, L and data not shown). These data suggest that *Hoxb4 *expression is preceded by the BMP signal-dependent dorsalization of the neural tube. It should be noted that the dorsalizing activity of exogenous BMP4 could be mediated by other members of the TGFβ super family whose function BMP4 can mimic. Because of the delay in the change in *Hoxb4 *following Smad6 electroporation, and the fact that no known Smad binding sites have been identified in *Hox *gene enhancer elements, it is likely that up-regulation of *Hoxb4 *is due to the dorsal feature of the neural tube induced by BMP signals, rather than the direct effect of activation of the BMP pathway.

## Discussion

Many previous studies have focused on vertical signals during the process of neural A-P patterning. While RA is likely to be the main signal derived from somites, it has been difficult to explain the neural A-P patterning process solely by RA/somite signals. The aim of this study is to shed light on new aspects other than the factor(s) derived from somites, that is, ectoderm-derived BMP/TGFβ signals and the subsequent acquired competence of the neural tube to respond to the somite/RA signal, together with planar signaling. This work also highlights a mechanism where RA and BMP4 act in a concerted manner to initiate neural *Hoxb4 *expression.

### Vertical and planar signaling

The classical idea of vertical and planar signaling has been proposed in studies of neural induction and patterning in amphibian embryos [44-46]. With regard to neural A-P patterning, Nieuwkoop proposed that extrinsic 'caudal influences' originate from the mesoderm [47] whereas others showed that planar signals alone can induce neural A-P pattern based on experiments with exogastrula embryos and Keller's explants [48]. Hence the role of vertical and planar signals in amphibian gastrulae remains debatable.

This work has focused on the process of neural patterning long after neural induction, revealed by up-regulation of *Hoxb4*. It has been demonstrated that vertical signals from somites are required but not solely responsible for the establishment of the *Hoxb4 *pattern (Fig. 3A–L). Additional signals emanating from more posterior tissues (neural tissue and/or somites) work in conjunction with the vertical signals to up-regulate *Hoxb4 *expression in anterior regions. These signals travel within the plane of the neural tube (planar signals), however, the source and identity of the planar signal remains to be determined. Based on the requirement of flanking somites for planar signaling (Fig. 4D, E), we suggest that somites provide not only instructive signals for *Hoxb4 *up-regulation but also permissive signals that assist in planar signaling.

Other studies have also suggested the existence of planar signals. This includes experiments where rhombomeres were transposed and incorporated at different A-P levels of the neuroepithelium. Induction of *Hox *genes was only observed in the grafted fragment of tissue providing the graft was perfectly incorporated into the host's neuroepithelium [15]. This suggested that the inducing signals are being transduced along the plane of the neural tube. In contrast, studies by others have discounted the possibility of planar signals. Analysis of the anterior extension of the *Hoxd4 *expression domain demonstrated that tissue continuity was not required in order for *Hoxd4 *expression to be established [35]. Implantation of a glass barrier in the neural tube of a 2 somite stage embryo posterior to the 2^nd ^somites did not prevent the extension of *Hoxd4 *expression, thus implying that planar signals are not necessary after the stage at which the glass barrier was placed. This experiment was reassessed in the present study using the *ex ovo *culture system with *Hoxb4 *as a marker (Fig. 4B and Additional file 1). The result showed a variable yet significant block of *Hoxb4 *induction at the anterior side of the barrier, suggesting that tissue continuity is indeed required during the normal course of *Hoxb4 *up-regulation.

The actual mode of action of the planar signal remains elusive. It is possible that one *Hoxb4*-expressing cell activates *Hoxb4 *expression in the cell(s) adjacent to it. This idea comes from an observation in *Xenopus *embryos, where injection of *Hoxb4 *mRNA into one blastomere causes induction of endogenous *Hoxb4 *expression outside of the injected lineage [49], suggesting that *Hoxb4 *expression is able to induce its expression in adjacent cells in a cell non-autonomous manner.

### The mode of action of the somite signal is influenced by the competence of the neural tube

This study, as well as previous work by others, has shown that in order to establish *Hoxb4 *expression in the neural tube, RA from the adjacent somites is required [12,30]. However, as seen from transverse sections of normal embryos (Fig. 5E–G), the somite, although adjacent to the neural tube, is not necessarily close to the dorsal side of the neural tube, which is where *Hoxb4 *is strongly expressed initially. This raises the question of how the neural tube exploits the RA/somite signal to initiate *Hoxb4 *expression. Somite rotation experiments showed no evidence of D-V difference in the strength of inducing ability in somites. This is in agreement with the expression pattern of *RALDH2*, which shows homogeneous expression along the D-V axis of the somites [21]. Our data suggest that RA is provided evenly at the dorsal and ventral sides of the neural tube, and it is the action of BMPs or other members of the TGFβ super family that may sensitize the dorsal neural tube to the RA signal, causing stronger *Hoxb4 *expression dorsally.

### The effect of D-V differences in establishing the regional specificity along the A-P axis

This work has suggested that BMP signaling is involved in rendering the neural tube competent to express *Hoxb4 *in response to RA or somite signals. Since BMP signaling is a specific feature of the dorsalization of the neural tube, this provides evidence that establishment of the *Hoxb4 *expression pattern, and hence establishment of A-P positional identity, is under the influence of D-V specific cellular characters, demonstrating interplay between the patterning of these two axes.

The phenomenon of A-P positional markers being initially up-regulated at the dorsal side of the neural tube is common in many *Hox *genes in the spinal cord and in *Krox20 *in rhombomere 5 [12,50]. However, it should be noted that the D-V difference might be only to influence the initial up-regulation and not to affect the expression domains of each *Hox *gene along the A-P axis. Genetically modified animals with affected D-V patterning in the neural tube, such as zebrafish embryos with compromised BMP signaling [51] and mouse embryos with deficiency in shh signals [52], exhibit correct A-P patterns. Therefore the dorsal specific features are, at most, to facilitate the establishment of the A-P patterns and not to give a clue for the correct A-P patterns. In fact, over-expression of BMP4 causes a ventral expansion of *Hoxb4 *only at the specific A-P level where *Hoxb4 *is normally expressed, and never anteriorly beyond the normal rhombomere 6/7 boundary (Figs. 7B, C; 8A, B). Thus the mechanism to prevent the anterior extension of expression domains is yet to be clarified.

### The effect of RA on *Hoxb4 *up-regulation

RA signaling is important not only in rhombomere patterning along the A-P axis but also in specifying the dorsal neural tube. Quail embryos deficient in RA exhibit a great loss of dorsal neural tube-specific markers such as *BMP4/7*, *Msx2 *and *Pax3/6/7 *[41]. However, RA does not appear to be sufficient for dorsalization of the neural tube. It is rather BMP4 that is responsible for dorsalization [38,53]. Hence it appears that RA is required to exert BMP4's dorsalizing activity. Another example of RA functioning in such a supporting manner is observed in the ventral neural tube during motor neuron differentiation. RA is required for the shh signal to induce *olig2 *in the ventral spinal cord [54,55]. Again, RA is not sufficient to induce *olig2 *in the absence of shh signaling; it is *shh *which induces *olig2*. Hence, in these contexts, RA acts as a factor to render the neural tube competent to other extrinsic signals such as shh and BMP4. In the case of *Hoxb4*, it has been shown that RA works directly as an inducer of *Hoxb4 *expression through an RARE in its enhancer [30]. In addition to this, the present study proposes that RA might also act to maintain the dorsal-specific domain that serves as an 'initial up-regulation area' for *Hoxb4*, which is likely induced by ectoderm-derived BMP4/TGFβ signals. Furthermore, our data showed distinct functions of RA and BMP signals: excess RA expands the *Hoxb4 *expressing domain anteriorly [12] but not ventrally (Fig. 7M), while excess BMP4 facilitates its ventral expansion. However, BMP4 cannot accomplish ventral expansion of *Hoxb4 *in the absence of RA (Fig. 7F, G). Hence RA and BMP4 are mutually required for *Hoxb4 *expression both in the dorsal domain as well as in the more ventral side of the neural tube.

## Conclusion

The mechanism of up-regulating *Hoxb4 *after neural tube formation was investigated. While vertical signals from somites are necessary for up-regulating *Hoxb4 *expression in the adjacent neural tube, these signals are not always instructive in nature; especially in the anterior-most region of the *Hoxb4 *expressing domain, the flanking somites do not sufficiently up-regulate its expression in the adjacent neural tube, yet they are necessary to provide permissive cues that allow the neural tube to respond to planar signals. *Hoxb4 *is initially up-regulated at the dorsal neural tube, with this up-regulation correlating with the dorsalized character of the neural tube. Moreover, somite/RA-dependent up-regulation of *Hoxb4 *is promoted by BMP signals. These data suggest that establishment of the *Hoxb4 *expression pattern, and hence establishment of A-P positional identity, is under the influence of D-V specific cellular characters, demonstrating interplay between the patterning of these two axes.

## Methods

### Chick explant culture

Fertilised chick eggs were incubated at 38.5°C until they reached the required somite stages. All dissections were performed in L-15 medium in a petri dish. The chick explant culture system utilises the two-drop collagen method: Collagen was extracted from rat tail by dissolving 1 g of tendon in 100 ml of 0.2% acetic acid, followed by dialysis against 0.1× DMEM (pH 4). The collagen solution was used at a concentration of 2 parts stock collagen to one part 0.1× DMEM. 270 μl of this working solution was then mixed with 30 μl of 10× DMEM. 5–10 μl of 1 M sodium bicarbonate was then added until the solution turned a pale orange colour. 20 μl drops of collagen were placed in each well of a 4-well plate (where each well measures 16 mm in diameter) and left to set for 30 minutes at 37°C with 5% CO_2_. Explants were placed on top of the first drop. A small groove was cut into the surface of the first collagen drop and the explant was placed carefully inside. In cases where somites should be removed from the neural tube, Dispase I (Roche) was used at 5 units/ml for no more than 3 minutes. Once somites and other surrounding tissues were removed, the neural tube was placed in a fresh drop of L15 thus removing any residual Dispase, this was followed by embedding the neural tube in the collagen drop. A second drop of collagen solution was added (approx. 15 μl) on top of the explant. The collagen was allowed to set as described above after which 400 μl of F-12 solution was added containing penicillin/streptomycin and 0.1% Mito serum extender (BD Biosciences). Explants were cultured for 24 hours at 37°C with 5% CO_2_, after which the explant was fixed with 4% paraformaldehyde in PBS for 15 minutes, gradually dehydrated to 100% methanol and stored at -20°C. *In situ *hybridization was performed in the same way as whole embryos as described previously [16].

### *In vivo *manipulations

For the purpose of placing a barrier across the axis *in vivo*, chick embryos at 2 or 10 somite stage were transferred to albumen-agar plates [36] and a piece of aluminium foil (approximately 300 mm × 900 mm) was placed at the level posterior to the 2^nd ^somite, separating all three germ layers into anterior and posterior sides. The embryos were incubated for 24–26 hours and processed for *in situ *hybridization.

Ablation experiments were performed *in ovo *using 3–4 somite stage embryos. Embryos were fixed at 10–12 somite stages and processed for *in situ *hybridization.

### Treatment of embryos with BMS493 and BMP4

A RA-receptor antagonist BMS493 was prepared following the same synthetic sequence described for related analogues [56]. Addition of the lithium derivative of 1-ethynylbenzene to 7-bromo-4,4-dimethyl-3,4-dihydronaphthalen-1-(2*H*)-one [56] to give the propargyl alcohol (82%), followed by dehydration with *p*-TsOH (83% yield), formylation (*n*-BuLi, then DMF, 76%) and Horner-Wadsworth-Emmons condensation with the anion of methyl 4-(diethylphosphonyl-methyl)-benzoate (*n*-BuLi, DMPU) afforded the entire arotinoid skeleton (89% yield). Saponification of the ester group afforded BMS493 (89% yield).

For treatment of embryos with BMS493, BMP4 or RA, embryos were dissected at 2 to 5 somite stages in L15 and cultured *ex ovo *[39] in optiMEM (GibcoBRL) with relevant compounds and/or proteins for 8–24 hours until they reached the 10–12 or 22 somite stages. BMS493 was used at the concentration of 4 μM. As the stock of BMS493 was dissolved in a 1:1 mix of DMSO and ethanol, control embryos were also cultured with the equivalent amount of DMSO and ethanol (0.004% each). Human recombinant BMP4 (rBMP4, R&D Systems) was used at a concentration of 60 ng/ml. BMP4 conditioned medium was obtained by transfecting HEK293 cells with a plasmid encoding BMP2/4 fusion for improved secretion of processed BMP4 [57]. Cells were incubated for two days after transfection with DMEM and 10% foetal calf serum before the conditioned medium was collected. Retinoic acid (*all-trans*, SIGMA) was used at the concentration of 500 nM.

### Embryo analyses

Flat mounting was performed after *in situ *hybridisation staining by dissecting the neural tube in 80% glycerol and opening it from the dorsal side. Vibratome sectioning (Fig. 5E–G, Fig. 6H, I, K, L) was performed by embedding embryos in 3–4% agarose and cutting at a thickness of 40 μm. Cryosectioning (Fig. 6C, F) was performed by embedding embryos in OCT compound (BDH) and cutting at a thickness of 13 μm.

## Authors' contributions

GSA and NI conceived and designed the experiments. GSA performed most of the embryonic work with substantial help from SH. SA and ARL synthesized BMS493. GSA, SH and NI wrote the manuscript with subsequent contributions from all authors. All authors read and approved the final manuscript.

## Supplementary Material

Additional file 1 **Barrier placement at the level posterior to the 2^nd ^somite inhibits up-regulation of *Hoxb4 *anterior to the barrier.** The data provided show all cases of the experiment shown in Fig. 4B. Click here for file

Additional file 2 **More posterior tissues provide stronger inductive signals compared to anterior tissues.** The data provided shows that the neural tube and somites from level 4 or 5 cause a stronger induction in the level 1 neural tube compared to those from more anterior levels such as levels 1–3. Click here for file

Additional file 3 **Number of cases of explants showing strong, weak or no expression of *Hoxb4 *after dissection of neural tube and somites at each somite level as shown in Figure**3A–L. The data provided represent the number of cases in the experiment shown in Fig. 3A–L. Click here for file

## References

[B1] Lumsden A, Krumlauf R (1996). Patterning the vertebrate neuraxis. Science.

[B2] Krumlauf R (1994). Hox genes in vertebrate development. Cell.

[B3] Gaunt SJ, Strachan L (1996). Temporal colinearity in expression of anterior Hox genes in developing chick embryos. Dev Dyn.

[B4] Wacker SA, Jansen HJ, McNulty CL, Houtzager E, Durston AJ (2004). Timed interactions between the Hox expressing non-organiser mesoderm and the Spemann organiser generate positional information during vertebrate gastrulation. Dev Biol.

[B5] Iimura T, Pourquie O (2006). Collinear activation of Hoxb genes during gastrulation is linked to mesoderm cell ingression. Nature.

[B6] Keynes R, Krumlauf R (1994). Hox genes and regionalization of the nervous system. Annu Rev Neurosci.

[B7] Hunt P, Krumlauf R (1992). Hox codes and positional specification in vertebrate embryonic axes. Annu Rev Cell Biol.

[B8] Krumlauf R, Marshall H, Studer M, Nonchev S, Sham MH, Lumsden A (1993). Hox homeobox genes and regionalisation of the nervous system. J Neurobiol.

[B9] McIntyre DC, Rakshit S, Yallowitz AR, Loken L, Jeannotte L, Capecchi MR, Wellik DM (2007). Hox patterning of the vertebrate rib cage. Development.

[B10] Pasqualetti M, Rijli FM (2001). Homeobox gene mutations and brain-stem developmental disorders: learning from knockout mice. Curr Opin Neurol.

[B11] Crawford M (1995). Transformations in null mutants of Hox genes: do they represent intercalary regenerates?. Bioessays.

[B12] Bel-Vialar S, Itasaki N, Krumlauf R (2002). Initiating Hox gene expression: in the early chick neural tube differential sensitivity to FGF and RA signaling subdivides the HoxB genes in two distinct groups. Development.

[B13] Oosterveen T, Niederreither K, Dolle P, Chambon P, Meijlink F, Deschamps J (2003). Retinoids regulate the anterior expression boundaries of 5' Hoxb genes in posterior hindbrain. Embo J.

[B14] Oosterveen T, Meijlink F, Deschamps J (2004). Expression of retinaldehyde dehydrogenase II and sequential activation of 5' Hoxb genes in the mouse caudal hindbrain. Gene Expr Patterns.

[B15] Grapin-Botton A, Bonnin MA, McNaughton LA, Krumlauf R, Le Douarin NM (1995). Plasticity of transposed rhombomeres: Hox gene induction is correlated with phenotypic modifications. Development.

[B16] Itasaki N, Sharpe J, Morrison A, Krumlauf R (1996). Reprogramming Hox expression in the vertebrate hindbrain: influence of paraxial mesoderm and rhombomere transposition. Neuron.

[B17] Grapin-Botton A, Bonnin MA, Le Douarin NM (1997). Hox gene induction in the neural tube depends on three parameters: competence, signal supply and paralogue group. Development.

[B18] Woo K, Fraser SE (1997). Specification of the zebrafish nervous system by nonaxial signals. Science.

[B19] Niederreither K, McCaffery P, Drager UC, Chambon P, Dolle P (1997). Restricted expression and retinoic acid-induced downregulation of the retinaldehyde dehydrogenase type 2 (RALDH-2) gene during mouse development. Mech Dev.

[B20] Swindell EC, Thaller C, Sockanathan S, Petkovich M, Jessell TM, Eichele G (1999). Complementary domains of retinoic acid production and degradation in the early chick embryo. Dev Biol.

[B21] Berggren K, McCaffery P, Drager U, Forehand CJ (1999). Differential distribution of retinoic acid synthesis in the chicken embryo as determined by immunolocalization of the retinoic acid synthetic enzyme, RALDH-2. Dev Biol.

[B22] Maden M, Sonneveld E, Saag PT van der, Gale E (1998). The distribution of endogenous retinoic acid in the chick embryo: implications for developmental mechanisms. Development.

[B23] Chambers D, Wilson L, Maden M, Lumsden A (2007). RALDH-independent generation of retinoic acid during vertebrate embryogenesis by CYP1B1. Development.

[B24] Rossant J, Zirngibl R, Cado D, Shago M, Giguere V (1991). Expression of a retinoic acid response element-hsplacZ transgene defines specific domains of transcriptional activity during mouse embryogenesis. Genes Dev.

[B25] Niederreither K, Subbarayan V, Dolle P, Chambon P (1999). Embryonic retinoic acid synthesis is essential for early mouse post-implantation development. Nat Genet.

[B26] Niederreither K, Vermot J, Schuhbaur B, Chambon P, Dolle P (2000). Retinoic acid synthesis and hindbrain patterning in the mouse embryo. Development.

[B27] Maden M, Gale E, Kostetskii I, Zile M (1996). Vitamin A-deficient quail embryos have half a hindbrain and other neural defects. Curr Biol.

[B28] Maden M (1999). Heads or tails? Retinoic acid will decide. Bioessays.

[B29] Gavalas A, Krumlauf R (2000). Retinoid signalling and hindbrain patterning. Curr Opin Genet Dev.

[B30] Gould A, Itasaki N, Krumlauf R (1998). Initiation of rhombomeric Hoxb4 expression requires induction by somites and a retinoid pathway. Neuron.

[B31] Subramanian V, Meyer BI, Gruss P (1995). Disruption of the murine homeobox gene Cdx1 affects axial skeletal identities by altering the mesodermal expression domains of Hox genes. Cell.

[B32] Isaacs HV, Pownall ME, Slack JM (1998). Regulation of Hox gene expression and posterior development by the Xenopus caudal homologue Xcad3. Embo J.

[B33] Shimizu T, Bae YK, Hibi M (2006). Cdx-Hox code controls competence for responding to Fgfs and retinoic acid in zebrafish neural tissue. Development.

[B34] Hamburger V, Hamilton HL (1951). A series of normal stages in the development of the chick embryo. Journal of Morphology.

[B35] Gaunt SJ, Strachan L (1994). Forward spreading in the establishment of a vertebrate Hox expression boundary: the expression domain separates into anterior and posterior zones, and the spread occurs across implanted glass barriers. Dev Dyn.

[B36] Chapman SC, Collignon J, Schoenwolf GC, Lumsden A (2001). Improved method for chick whole-embryo culture using a filter paper carrier. Dev Dyn.

[B37] Timmer JR, Wang C, Niswander L (2002). BMP signaling patterns the dorsal and intermediate neural tube via regulation of homeobox and helix-loop-helix transcription factors. Development.

[B38] Liem KF, Tremml G, Roelink H, Jessell TM (1995). Dorsal differentiation of neural plate cells induced by BMP-mediated signals from epidermal ectoderm. Cell.

[B39] Connolly D, McNaughton LA, Krumlauf R, Cooke J (1995). Improved in vitro development of the chick embryo using roller-tube culture. Trends Genet.

[B40] Germain P, Iyer J, Zechel C, Gronemeyer H (2002). Co-regulator recruitment and the mechanism of retinoic acid receptor synergy. Nature.

[B41] Wilson L, Gale E, Chambers D, Maden M (2004). Retinoic acid and the control of dorsoventral patterning in the avian spinal cord. Dev Biol.

[B42] Wilson L, Maden M (2005). The mechanisms of dorsoventral patterning in the vertebrate neural tube. Dev Biol.

[B43] Imamura T, Takase M, Nishihara A, Oeda E, Hanai J, Kawabata M, Miyazono K (1997). Smad6 inhibits signalling by the TGF-beta superfamily. Nature.

[B44] Doniach T (1993). Planar and vertical induction of anteroposterior pattern during the development of the amphibian central nervous system. J Neurobiol.

[B45] Ruiz i Altaba A (1992). Planar and vertical signals in the induction and patterning of the Xenopus nervous system. Development.

[B46] Grunz H, Schuren C, Richter K (1995). The role of vertical and planar signals during the early steps of neural induction. Int J Dev Biol.

[B47] Nieuwkoop PD, Others (1952). Activation and organization of the central nervous system in amphibians. J Exp Zool.

[B48] Doniach T, Phillips CR, Gerhart JC (1992). Planar induction of anteroposterior pattern in the developing central nervous system of Xenopus laevis. Science.

[B49] Hooiveld MH, Morgan R, in der Rieden P, Houtzager E, Pannese M, Damen K, Boncinelli E, Durston AJ (1999). Novel interactions between vertebrate Hox genes. Int J Dev Biol.

[B50] Irving C, Nieto MA, DasGupta R, Charnay P, Wilkinson DG (1996). Progressive spatial restriction of Sek-1 and Krox-20 gene expression during hindbrain segmentation. Dev Biol.

[B51] Barth KA, Kishimoto Y, Rohr KB, Seydler C, Schulte-Merker S, Wilson SW (1999). Bmp activity establishes a gradient of positional information throughout the entire neural plate. Development.

[B52] Lebel M, Mo R, Shimamura K, Hui CC (2006). Gli2 and Gli3 play distinct roles in the dorsoventral patterning of the mouse hindbrain. Dev Biol.

[B53] Liem KF, Tremml G, Jessell TM (1997). A role for the roof plate and its resident TGFbeta-related proteins in neuronal patterning in the dorsal spinal cord. Cell.

[B54] Novitch BG, Wichterle H, Jessell TM, Sockanathan S (2003). A requirement for retinoic acid-mediated transcriptional activation in ventral neural patterning and motor neuron specification. Neuron.

[B55] Diez del Corral R, Olivera-Martinez I, Goriely A, Gale E, Maden M, Storey K (2003). Opposing FGF and retinoid pathways control ventral neural pattern, neuronal differentiation, and segmentation during body axis extension. Neuron.

[B56] Vuligonda V, Standeven AM, Escobar M, Chandraratna RA (1999). A new class of potent RAR antagonists: dihydroanthracenyl, benzochromenyl and benzothiochromenyl retinoids. Bioorg Med Chem Lett.

[B57] Liem KF, Jessell TM, Briscoe J (2000). Regulation of the neural patterning activity of sonic hedgehog by secreted BMP inhibitors expressed by notochord and somites. Development.

