# Supplementary material for: Regulation of Hoxb4 induction after neurulation by somite signal and neural competence
Source: BMC Dev Biol. 2009 Feb 25;9:17. doi: 10.1186/1471-213X-9-17 (PMC2667173; doi:10.1186/1471-213X-9-17)
Supplement: Additional file 1 — Barrier placement at the level posterior to the 2nd somite inhibits up-regulation of Hoxb4 anterior to the barrier. The data provided show all cases of the experiment shown in Fig. 4B. [file 1471-213X-9-17-S1.pdf]

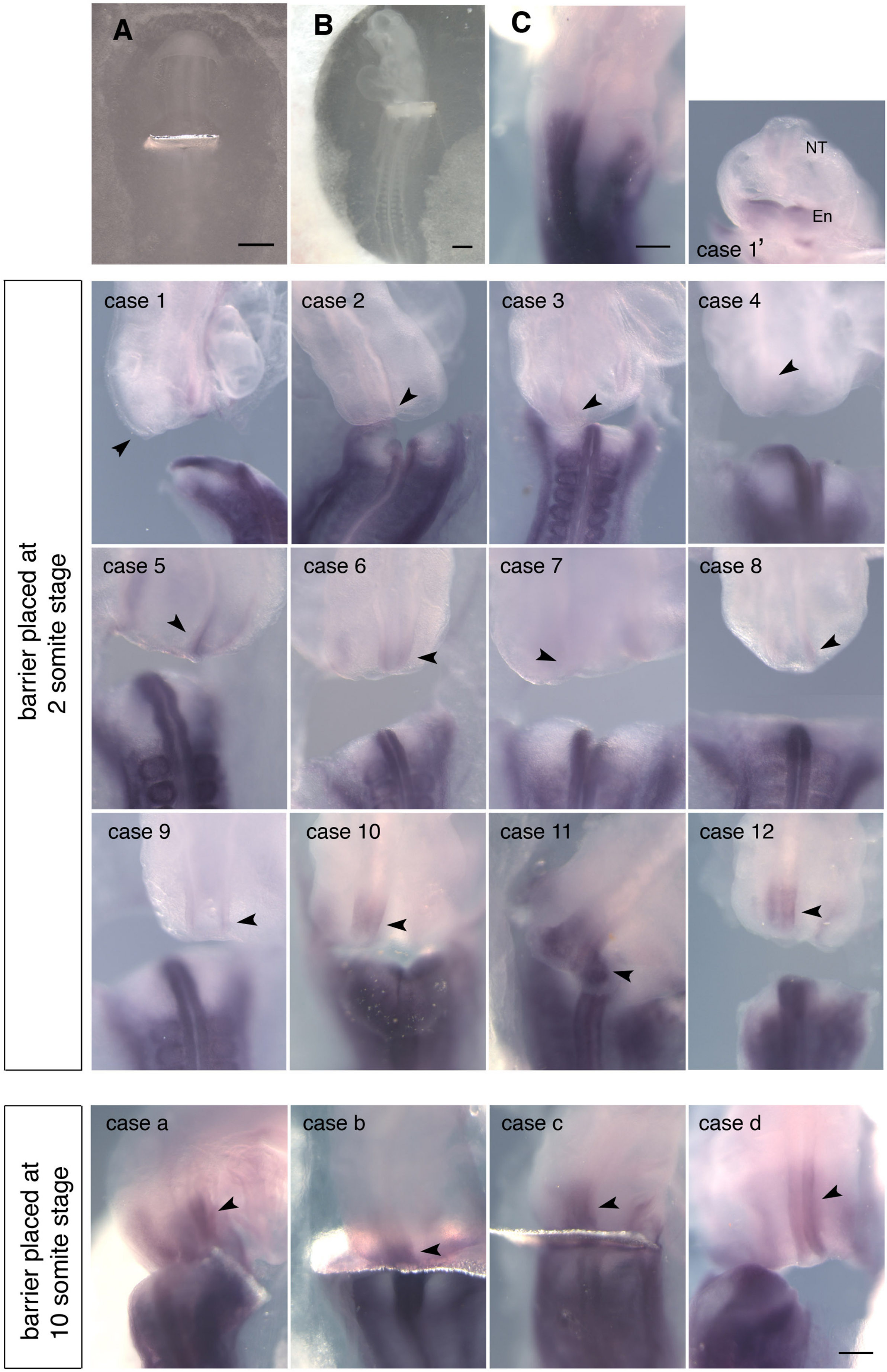
A
B
C
NT
En
case 1'
barrier placed at 2 somite stage
case 1
case 2
case 3
case 4
case 5
case 6
case 7
case 8
case 9
case 10
case 11
case 12
barrier placed at 10 somite stage
case a
case b
case c
case d

Additional file 1

Barrier placement at the level posterior to the 2nd somite inhibits up-regulation of *Hoxb4* anterior to the barrier

Embryos at the 2 somite stage were cultured *ex ovo* on albumen-agar plates and foil barriers were placed posterior to the 2nd somite (future somite 2/3 boundary). Embryos were incubated for 24 hours and processed for *in situ* hybridization for *Hoxb4*. As a control, embryos without barrier placement were processed in parallel.
(A) and (B) show examples of embryos placed with a barrier, before (A) and after (B) the 24 hour incubation. Scale bars in (A) and (B) indicate 500 µm. (C) is a control embryo cultured *ex ovo* without barrier, stained for *Hoxb4* (21 somite stage). Scale bar, 200 µm.
13 embryos were obtained by three independent experiments and one under-developed embryo (15 somite stage after the 24 hour culture) was omitted from further analyses. All analysed cases (case 1-12) showed complete separation of the neural tube and somites. Case 9 is also shown in Fig 4B. The top right panel (case 1') is a view of the transverse surface of the anterior side of the barrier in case 1, showing that *Hoxb4* is positive in the pharyngeal endoderm (En) but not in the neural tube (NT).

The results were evaluated by comparing the *Hoxb4 in situ* staining on the anterior side of the barrier to that on the posterior side.

negative:	No expression is seen.
faint:	Faint expression is seen.
weak:	Expression is significant, yet not as strong as the posterior side of the barrier.
strong:	Expression is as strong as the posterior side of the barrier.

Nine out of twelve cases show faint or no expression of *Hoxb4* in the neural tube anterior to the barrier.

As an additional control, a barrier was placed in 5 embryos at the 10 somite stage and the embryos were incubated until they reached the similar developmental stages to the ones in the above set of experiments. One embryo in which the neural tube was not completely divided by the barrier was omitted from further analyses. All four cases are shown (case a-d). In two out of four cases (a and c), *Hoxb4* expression at the anterior side of the barrier was as strong as the posterior side.

The neural tube anterior to the barrier is indicated by arrowheads in each case.
Scale bar; 200 µm.
The summary of the result is shown in below tables.

Barrier placed at the 2 somite stage

Case#	Analyzed stage (somite number)	*Hoxb4* expression at the anterior side of the barrier
1	21	negative
2	18	negative
3	19	faint
4	22	negative
5	17	faint
6	18	faint
7	19	negative
8	19	faint
9	19	faint
10	20	weak
11	22	strong
12	19	weak

Barrier placed at 10 somite stage

Case#	Analyzed stage (somite number)	*Hoxb4* expression at the anterior side of the barrier
a	22	strong
b	18	weak
c	19	strong
d	23	weak
