# Supplementary figures and images for: Regulation of Hoxb4 induction after neurulation by somite signal and neural competence
Source: BMC Dev Biol. 2009 Feb 25;9:17. doi: 10.1186/1471-213X-9-17 (PMC2667173; doi:10.1186/1471-213X-9-17)

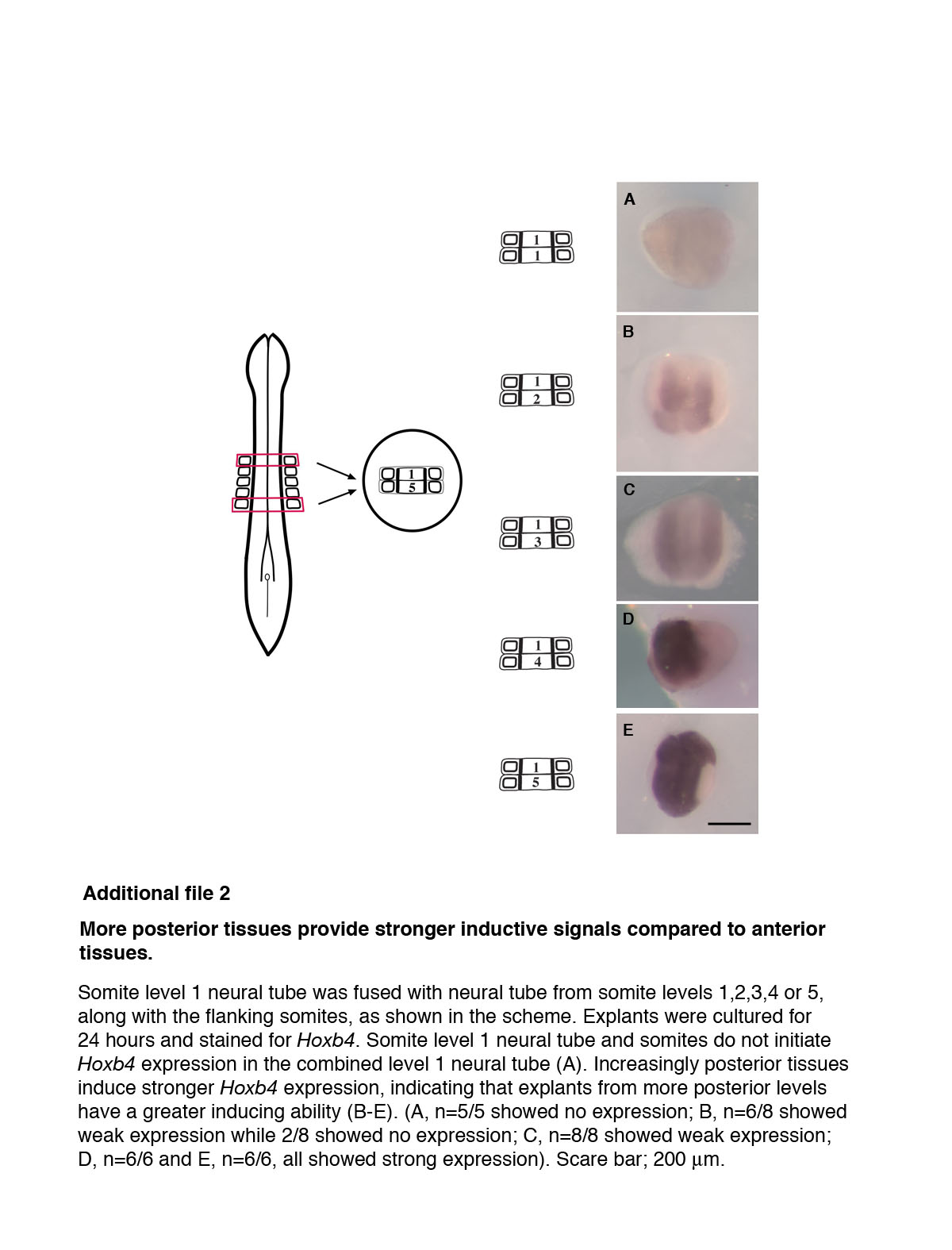

Supplement: Additional file 2 — More posterior tissues provide stronger inductive signals compared to anterior tissues. The data provided shows that the neural tube and somites from level 4 or 5 cause a stronger induction in the level 1 neural tube compared to those from more anterior levels such as levels 1–3. [file 1471-213X-9-17-S2.jpeg]
