# Supplementary material for: Regulation of Hoxb4 induction after neurulation by somite signal and neural competence
Source: BMC Dev Biol. 2009 Feb 25;9:17. doi: 10.1186/1471-213X-9-17 (PMC2667173; doi:10.1186/1471-213X-9-17)
Supplement: Additional file 3 — Number of cases of explants showing strong, weak or no expression of Hoxb4 after dissection of neural tube and somites at each somite level as shown in Figure3A–L. The data provided represent the number of cases in the experiment shown in Fig. 3A–L. [file 1471-213X-9-17-S3.pdf]

Additional file 3.

Number of cases of explants showing strong, weak or no expression of *Hoxb4* after dissection of neural tube and somites at each somite level as shown in Figure 3A-L.

Stage of dissection	Somite level	No up-regulation	Weak up-regulation	Strong up-regulation
3 somite stage	1	4/4	-	-
	2	1/3	2/3	-
	3	-	-	3/3
4 somite stage	1	4/5	1/5	-
	2	-	6/7	1/7
	3	-	5/6	1/6
	4	-	-	6/6
5 somite stage	1	5/6	1/6	-
	2	1/8	7/8	-
	3	-	6/8	2/8
	4	-	-	5/5
	5	-	-	8/8
